# A dynamic integrated scheduling method based on hierarchical planning for heterogeneous AGV fleets in warehouses

**DOI:** 10.3389/fnbot.2022.1053067

**Published:** 2023-01-09

**Authors:** Enze Hu, Jianjun He, Shuai Shen

**Affiliations:** The School of Automation, Central South University, Changsha, China

**Keywords:** automated guided vehicles, dynamic integrated scheduling, task assignment, path planning, hierarchical planning, hybrid discrete state transition algorithm

## Abstract

In modern industrial warehouses, heterogeneous and flexible fleets of automated guided vehicles (AGVs) are widely used to improve transport efficiency. However, as their scale and limit of battery capacity increase, the complexity of dynamic scheduling also increases dramatically. The problem is to assign tasks and determine detailed paths to AGVs to keep the multi-AGV system running efficiently and sustainedly. In this context, a mixed-integer linear programming (MILP) model is formulated. A hierarchical planning method is used, which decomposes the integrated problem into two levels: the upper-level task-assignment problem and the lower-level path-planning problem. A hybrid discrete state transition algorithm (HDSTA) based on an elite solution set and the Tabu List method is proposed to solve the dynamic scheduling problem to minimize the sum of the costs of requests and the tardiness costs of conflicts for the overall system. The efficacy of our method is investigated by computational experiments using real-world data.

## 1. Introduction

With the development in automation technology, AGVs as an important component of the modern warehouse logistics system is getting increased attention because of their accuracy, flexibility, and efficiency. More recently, heterogeneous AGV fleets are rapidly being adopted by industrial instances to perform different material handling tasks, where each vehicle has specific capabilities (e.g., pallet truck AGVs can tow loads, while backpack AGVs can lift loads). The minimization of travel costs is the most important objective of dynamic scheduling pursued in practice, which is affected by various decisions such as task assignment (i.e., assigning and sequencing tasks to AGVs), path planning (i.e., selecting optimal paths taken by each vehicle to reach the destination), and conflict management (i.e., avoiding conflicts between AGVs). These subproblems are interdependent; therefore, optimizing scheduling problems sequentially may yield a suboptimal performance of the overall AGV system (Maza and Castagna, 2005).

An example of a warehouse trying to implement an automated material handling system using a heterogeneous AGV fleet is Trucking Company (TC), which is a high-tech listed company in Changsha, China. Currently, the vehicle management system used in TC relies on prepackaged software provided by AGV manufacturers. However, such software packages are not applicable to a heterogeneous AGV fleet and cannot handle dynamic problems such as the addition of new tasks and charging requests for AGVs. In addition, the optimal task assignment scheme may cause more traffic jams during path planning, and an evaluation index needs to be quantified and established for the delay time caused as a result of the waiting and detour strategy of the AGVs. As the configuration cost increases, a real-time and efficient integrated scheduling method becomes important in improving the economic performance of the warehouse. In this study, we focus on a dynamic integrated scheduling problem for heterogeneous AGVs with battery constraints.

Motivated by our collaboration with the TC, the main novelty of our problem setting in contrast to the existing literature is constituted by the combination of the following features. First, we specifically focused on solving the scheduling problems right on time, whereas the methods in most studies consume unreasonable computational effort, in particular, some exact methods (Schiffer and Walther, 2017; Ma et al., 2020; Singh et al., 2022). Second, the AGVs considered in this study are heterogeneous in terms of battery management, travel speed, and capabilities to perform transportation of different types of materials, which increases the complexity of the problem. Third, we simultaneously considered joint task assignments, path planning, and conflicts that reduce the problems of the AGV system. We aimed to make decisions on optimizing the overall AGV system performance rather than successively solving each subproblem. Our main contributions are summarized as follows:

First, we developed a mixed-integer linear programming (MILP) model for analyzing the scheduling of multi-AGVs, which combines both task assignment and path planning in automated warehouses. The model captures conflicts between a heterogeneous set of AGV fleets, allowing for scheduling according to the uncertain environment. The objective is to minimize the sum of the costs of requests and costs of conflicts. Constraints are also formulated to cope with features of capacity and battery management.

Second, the hierarchical planning method was used to decompose the complex and integrated scheduling problem. We propose a hybrid discrete state transition algorithm (HDSTA) considering the two-layer problems based on incorporating an elite solution set and the Tabu Search to find the optimal solution for the overall system instead of optimal solutions for each independent problem. Although our model is stylized for warehouses, the method can be applied to other applications such as flexible manufacturing systems and automated container terminals.

Third, we present the concept of a path expert database and its generation methods. The selection procedure based on a preset database is established for real-time path planning, which provides the foundation for dynamic scheduling.

Finally, numerical experiments are performed to validate the model according to the real-world data of warehouses in Changsha, China. Our approach is shown to yield approximate optimal solutions for AGV scheduling and path planning within a reasonable timeframe.

The remainder of this article is organized as follows. Relevant literature on the scheduling of multi-AGVs is discussed in the “Literature review” section. Problem description and the MILP model are formally established in the “Dynamic scheduling system and problem description” section. The “Hierarchical planning method” section presents the hierarchical planning method and introduces the proposed HDSTA and the selection procedure based on the path expert database. The “Computational experiments” section reports the experiments conducted to test the proposed method. Finally, conclusions and several future research directions are discussed in the “Conclusion” section.

## 2. Literature review

AGV scheduling can directly determine the efficiency and the cost of the overall transport system and therefore high attention is paid by researchers or manufacturing enterprises. Fazlollahtabar and Saidi-Mehrabad (2015) presented a literature review and divided AGV scheduling into three subproblems, task assignment, path planning, and collision avoidance. Many studies applied various methods, such as exact methods, heuristics, and meta-heuristics, to treat the subproblems separately or simultaneously.

As for exact methods, Desaulniers et al. (2003) designed an exact method including three algorithms (greedy search, column generation, and branch cutting), which enables solving the scheduling problem for four vehicles. Nishi et al. (2011) addressed a Lagrangian relaxation and cut scheme under the bilevel decomposition framework to optimize simultaneous task assignments and conflict-free routing problems. Fazlollahtabar and Hassanli (2018) presented a modified network simplex algorithm for blocking a scheduling problem in the manufacturing system. Nevertheless, because of the non-deterministic polynomial-time (NP)-hard nature of the scheduling problems, the exact method is only suitable for instances of small-scale problems.

For large-scale complex real-world problems, heuristics or metaheuristics are mainly adopted. Li et al. (2019) proposed an improved harmony search algorithm to improve the AGV scheduling rate, which can obtain the best harmony by considering the rate change. Zhang et al. (2019) proposed a genetic algorithm and a hybrid-load AGV scheduling model to reduce the total cost of the logistics system, which was successfully applied to a mixed-model automobile assembly line. Abderrahim et al. (2020) used a variable neighborhood search algorithm to assign tasks in a manufacturing shop based on a vehicle manufacturing facility to minimize the maximum completion time. Zhang et al. (2022) proposed an improved iterated greedy algorithm to solve the AGV dispatching problem to minimize the total transportation cost of the matrix manufacturing workshop. In addition, many other meta-heuristics were also used in scheduling problems, such as the simulated annealing algorithm (Lu and Wang, 2019), the two-stage ant colony algorithm (Hamzeei et al., 2013), the evolutionary algorithm (Saidi-Mehrabad et al., 2015), and the particle swarm optimization algorithm (Gen et al., 2017). In the above literature, a common feature of the problems studied is that all the task information are stable and obtained in advance, and then, an analytical model was established and the problems are solved with a heuristic or meta-heuristic algorithm. Nevertheless, in a real-world instance, it is unrealistic to obtain all the task information in advance, while many uncertainties (e.g., urgent tasks and task rework) exist under dynamic and complex environments (Zhang et al., 2017). Therefore, the static scheduling method is insufficient for the complicated real-world industrial environment.

In recent years, with the development of IoT technology, many researchers focused on the dynamic scheduling problem. Li et al. (2020) proposed a multi-vehicle AGV scheduling mechanism for simulating multicustomer demands in an intelligent warehouse system. Mourtzis et al. introduced a cloud-based cyber-physical system with the help of IoT to achieve adaptive shop floor scheduling and condition-based maintenance. Umar et al. (2015) proposed an improved hybrid genetic algorithm method for dynamic scheduling that considers dispatching and conflict-free routing problems of AGVs under a flexible workshop environment. Lyu et al. (2019) presented an improved genetic algorithm combined with the Dijkstra algorithm considering time windows to solve the problems of optimal numbers, shortest transportation time, and conflict-free routing in the path planning process. Qiuyun et al. (2021) improved the particle swarm optimization algorithm to obtain the shortest transportation time for the AGV path planning problem of a one-line production line in manufacturing. Guo et al. (2020) studied the acceleration control method and the AGV priority determination method to improve the negotiation of AGVs that implement conflict-free path planning. Nevertheless, these researchers ignored the influence of not only the case of AGV heterogeneity but also battery management.

Through the review of the above literature, there have been no studies on dynamic integrated scheduling in warehouses for a heterogeneous set of AGV fleets with battery constraints. Therefore, a novel scheduling approach for AGVs is in high demand. In this study, we propose an HDSTA under a hierarchical planning framework to solve the complex problem, which is a kind of intelligent optimization algorithm with good global search capability and convergence property, considering the solution as a state and the update of the solution as a state transition process. Thus, we evaluated the proposed method with an industrial case study finally.

## 3. Dynamic scheduling system and problem description

In this section, a dynamic scheduling system for AGVs is proposed, which is based on a control system using inertial navigation guidance and QR codes. The information service is implemented by network and wireless routers. The integrated scheduling problems of heterogeneous AGVs with battery constraints in the AGV system are described and formulated while the conflict problem is highlighted.

### 3.1. Dynamic schedule system

**Figure 1:**
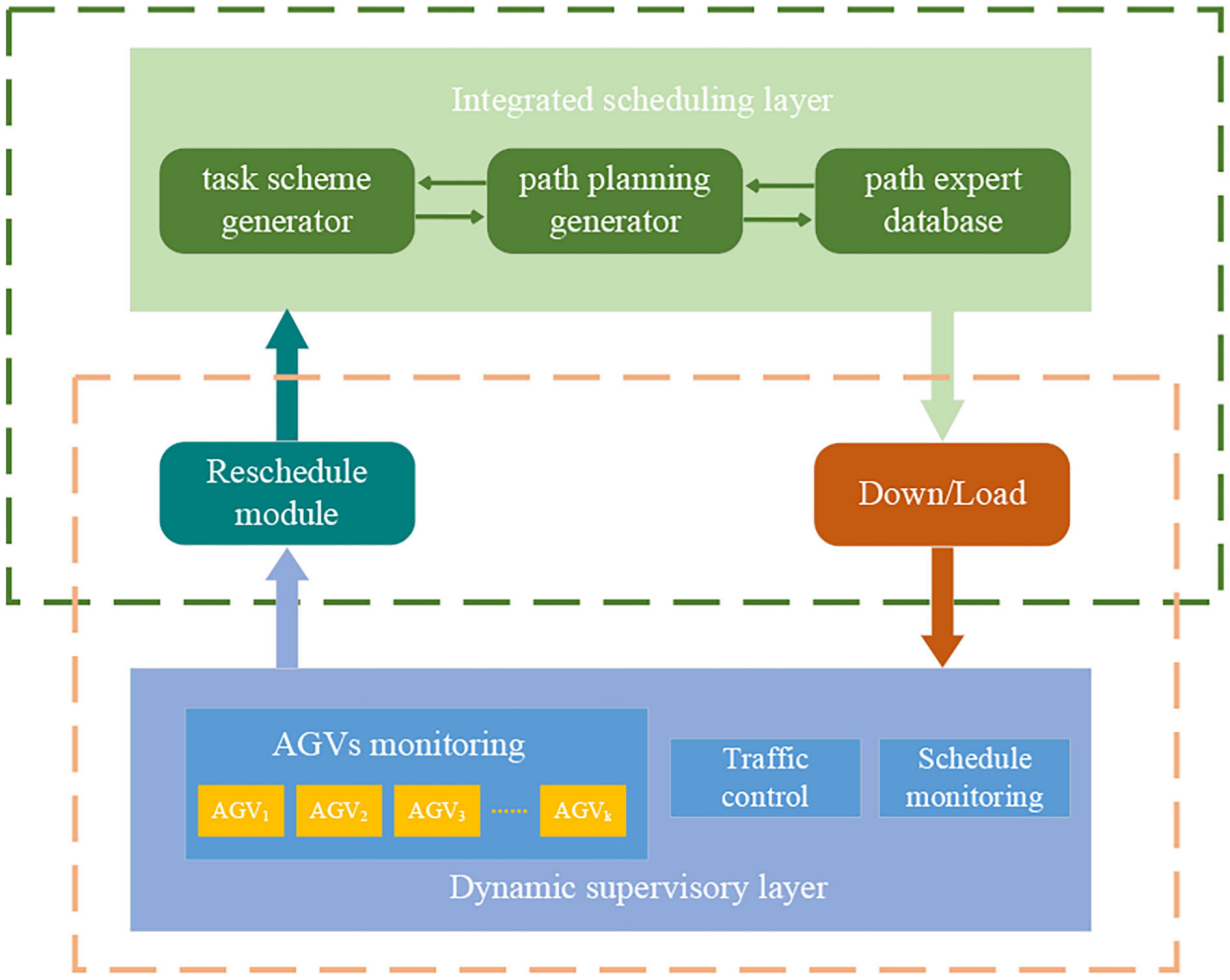
Dynamic scheduling system.

The overall architecture of the dynamic scheduling system is presented in Figure 1. The dynamic supervisory layer provides real-time information about AGVs and the current schedule. The AGV monitoring system is responsible for managing the AGVs in terms of recognition, positioning, motor control, and battery level. The schedule monitoring system is responsible for receiving new tasks while monitoring the implementation of the current schedule and requesting a new schedule as a result of a change of tasks. The rescheduled module initialization harmonizes additional parameters with the running schedule that includes active AGVs, new tasks, completed tasks, and in-process tasks.

The integrated scheduling layer is responsible for determining the task assignment/sequence and path planning, which is more complex because of the consideration of conflict avoidance. AGV movement on warehouse layout is a multigraph problem, in that there are various parallel paths between the presorting stations. The path expert database is established in the offline stage, which can be regarded as a dataset containing warehouse layout information and the candidate elite paths sets between each presorting station. Accessory equipment such as sensors are equipped which enables AGVs to detect moving objects by hardware and avoid collision by preprocessed combination strategies of traffic regulations (e.g., stop and wait for the higher priority AGV to pass first or move around the conflict location). Conflicts can also be reduced by combining and changing the task assignment/sequence if it cannot be solved separately by path selection. However, the delay time as a result of the waiting and detour strategy of AGVs needs to be quantified and reduced. The schedule contains task assignment/sequence and path planning, which are generated by the task scheme generator and the path planning generator. The generated schedule is downloaded for execution by the system.

In a dynamic system, the assumptions considered are as follows: (1) loading and unloading times are fixed; (2) the AGVs move in four directions; and (3) the positioning deviation of the AGVs is negligible.

### 3.2. Problem statement

This study considers a real-world industry case of the TC where the goal is to have continuous material handling without human interference. Each transport process of the AGVs is composed of pickup travel, loading, delivery travel, and unloading. The layout of the warehouse is modeled as a multigraph, *G* = (*N, E*), where *N* = {1, 2, …, *n*} is a set of all the nodes. Let *C* ⊂ *N* denote the set of charging stations and *X* ⊂ *N* denote the set of presorting stations, respectively. Moreover, *E* = {(*i, j, p*):*i, j* ∈ *N, i* ≠ *j*} denotes the set of arcs between every node pair. The *p*^*th*^ path between the nodes *i* and *j* is represented by (*i, j, p*) ∈ *E*. Parallel paths are stored in the path expert database *N*_*s*_ which is established in the offline stage. If there is a collision between a pair of current paths, a path parallel to one of this pair of current paths can be used to replace this path for avoiding conflict.

In our problem setting, a set of transport tasks *T* are serviced by a set of heterogeneous AGVs *K*, and each task *r* ∈ *T* contains a pickup node and a delivery node which are denoted by *u*_*r*_ ∈ *X* and *d*_*r*_ ∈ *X*, respectively. Besides transport task requests, a set of charging requests is denoted by *B* = {1, 2, …, |*B*|}, where |*B*| = |*C*| • |*K*| is the upper bound which is sufficiently large and *C* represents a set of charging stations. For each charging request *b* ∈ *B*, the pickup node and the delivery node are the same. The AGV makes a start instruction at the origin station *s* and each request contains only one delivery node. A termination instruction will be issued when the AGV reaches the terminal station *e*. Multiple request sets are defined by *R* = *T* ∪ *B, R*_*s*_ = *R* ∪ {*s*}, *R*_*e*_ = *R* ∪ {*e*}, and *R*_*se*_ = *R* ∪ {*s*} ∪ {*e*}.

We considered battery constraints and the maximum and minimum battery levels for each AGV, where *k* ∈ *K* are denoted by $b_{h}^{k}$ and $b_{l}^{k}$, respectively. Before performing a new task, the battery level *b*_*k*_ of each AGV needs to be above the minimum threshold $b_{l}^{k}$. The AGV is not allowed to access the charging facilities while traveling with a load. AGVs are required to complete the current task with the first priority before going to the charging station. The discharging rate of AGV *k* ∈ *K* is represented by *d*_*k*_, while each AGV has a unit time travel cost of *c*_*k*_.

As previously mentioned, AGVs are heterogeneous in terms of their capabilities to perform the transportation of different types of materials. Let $C_{r}^{T}$ denote a set of capability requirements for each task, and each AGV has a specific capability $C_{k}^{K}$. The task *r* ∈ *T* is only able to be performed by AGV *k* ∈ *K* if $C_{r}^{T}\subseteq C_{k}^{K}$ holds to ensure that each task is performed by an AGV with corresponding handling capacity. For example, the tasks of lifting loads need to be performed by backpack AGVs while the pallet truck AGVs are only able to tow loads.

The path from the pickup node to the delivery node *r* is denoted by *P*_*r*_, and the path from the delivery node of task *r* to the pickup node of task *r*′ is denoted by $P_{rr^{{\;}^{\prime }}}$. The AGV has its specific forward speed and velocity of rotation, respectively. For each path *p* ∈ *P*_*r*_, the travel time of AGV *k* is denoted by $T_{rp}^{k}$, and for each path $p\in P_{rr^{{\;}^{\prime }}}$, the travel time of AGV *k* is denoted by $T_{rr^{{\;}^{\prime }}p}^{k}$. In addition, the conflict-free path is optional during path planning because collisions can be prevented by traffic regulations. A delay time returns when an AGV follows a waiting and detour strategy to avoid a collision. When the path *p* ∈ *P*_*r*_ of AGV *k* conflicts with the path *q* ∈ *P*_*m*_ of AGV *g*, the delay time of AGV *k* on the path *p* ∈ *P*_*r*_ is defined by $\Phi_{rkp}^{mgq}\left(z_{rk}^{u},z_{mg}^{u}\right)$, where $z_{rk}^{u}$ represents the time for AGV *k* to arrive at the pickup node of request *r* and $z_{mg}^{u}$ represents the time for AGV *g* to arrive at the pickup node of request *m*, respectively. When the path *p* ∈ *P*_*r*_ of AGV *k* conflicts with the path $q\in P_{mm^{{\;}^{\prime }}}$ of AGV *g*, the delay time of AGV *k* on the path *p* ∈ *P*_*r*_ is defined by $\Phi_{rkp}^{mm^{{\;}^{\prime }}gq}\left(z_{rk}^{u},z_{mg}^{d}\right)$, where $z_{mg}^{d}$ represents the time for AGV *g* to arrive at the delivery node of request *m*.

### 3.3. Mixed-integer linear programming model

In this section, we formulate a mathematical model based on the problem description, which is an improvement from the findings of Dang et al. (2021) and Singh et al. (2022). Decision variables are introduced as follows:

$x_{rk}^{p}$: binary variable equal to 1 if AGV *k* ∈ *K* travels from the pickup node to the delivery node of request *r* ∈ *R* using *p* ∈ *P*_*r*_ or 0 otherwise
$y_{rr^{\prime }k}^{p}$: binary variable equal to 1 if AGV *k* ∈ *K* travels from the delivery node of request *r* ∈ *R*_*s*_ to the pickup node of request $r^{{\;}^{\prime }}\in R_{e}$ using ${p\in P}_{rr^{{\;}^{\prime }}}$ or 0 otherwise
$z_{rk}^{u}$: time of AGV *k* at the pickup node *u*_*r*_ of request *r* ∈ *R*_*e*_; $z_{ek}^{u}$ is the termination time of AGV *k*
$z_{rk}^{d}$: time of AGV *k* at the delivery node *d*_*r*_ of request *r* ∈ *R*_*s*_; $z_{sk}^{d}$ is the start time of AGV *k*
$\lambda_{rk}^{u}$: percent amount of battery discharge of AGV *k* at the pickup node *u*_*r*_ of request *r* ∈ *R*_*e*_
$\lambda_{rk}^{d}$: percent amount of battery discharge of AGV *k* at the delivery node *d*_*r*_ of request *r* ∈ *R*_*s*_

The mathematical model of the described problem is presented as follows:

The objective function *f* is to minimize the sum of the costs of requests and the tardiness costs of conflicts as the cost of each AGV is directly proportional to its travel time.


$$\begin{array}{l} f=\min\overset{\;}{\underset{k\in K}{\sum }}\overset{\;}{\underset{k}{c}}\left(\overset{u}{\underset{ek}{z}}-\overset{d}{\underset{sk}{z}}\right) \end{array}$$

(1) $$\begin{array}{l} \underset{k\in K}{\sum }\underset{p\in p_{r}}{\sum }{{\text{x}}^{p}}_{rk}=1\forall r\in T \end{array}$$

(2) $$\begin{array}{lll} \underset{k\in K}{\sum }\underset{p\in p_{r}}{\sum }{{\text{x}}^{p}}_{rk} & \leq & 1\forall r\in B \end{array}$$

(3) $$\begin{array}{l} {x^{p}}_{rk}=0\;\forall p\in P_{r},\forall r\in T,\forall k\in K,C_{r}^{T}⊈C_{k}^{K} \end{array}$$

(4) $$\begin{array}{l} {y^{p}}_{rrk}=0\forall r\in R,\forall k\in K \end{array}$$

(5) $$\begin{array}{l} \underset{r\in R_{s}}{\sum }\underset{p\in p_{rr^{\prime }}}{\sum }{y^{p}}_{rr^{\prime }k}=\underset{p\in p_{r^{\prime }}}{\sum }{x^{p}}_{r^{\prime }k}\forall r^{\prime }\in R,\forall k\in K \end{array}$$

(6) $$\begin{array}{l} \underset{p\in p_{r}}{\sum }{x^{p}}_{rk}=\underset{r^{\prime }\in R_{e}}{\sum }\underset{p\in p_{rr^{\prime }}}{\sum }{y^{p}}_{rr^{\prime }k}\forall r\in R,\forall k\in K \end{array}$$

$$\begin{array}{l} z_{rk}^{u}+T_{rk}^{p}+\underset{g\in K}{\sum }\underset{m\in T}{\sum }\underset{q\in p_{m}}{\sum }\Phi_{rkp}^{mgq}\left(z_{rk}^{u},z_{mg}^{u}\right){x^{q}}_{mg}\\ +\underset{g\in K}{\sum }\underset{m\in R_{s}}{\sum }\underset{m^{\prime }\in R_{e}}{\sum }\underset{q\in p_{mm^{\prime }}}{\sum }\Phi_{rkp}^{mm^{\prime }gq}\left(z_{rk}^{u},z_{mg}^{d}\right)\\ {}*{y^{q}}_{mm^{\prime }g}-M\left(1-{x^{p}}_{rk}\right)\leq z_{rk}^{d}\forall p\in P_{r}, \end{array}$$

(7) $$\begin{array}{l} \forall r\in T,\forall k\in K \end{array}$$

$$\begin{array}{l} z_{rk}^{d}+T_{rr^{\prime }k}^{p}+\underset{g\in K}{\sum }\underset{m\in T}{\sum }\underset{q\in p_{m}}{\sum }\Phi_{rr^{\prime }kp}^{mgq}\left(z_{rk}^{d},z_{mg}^{u}\right){x^{q}}_{mg}\\ +\underset{g\in K}{\sum }\underset{m\in R_{s}}{\sum }\underset{m^{\prime }\in R_{e}}{\sum }\underset{q\in p_{mm^{\prime }}}{\sum }\Phi_{rr^{\prime }kp}^{mm^{\prime }gq}\left(z_{rk}^{d},z_{mg}^{d}\right)\\ {}*{y^{q}}_{mm^{\prime }g}-M\left(1-{y^{p}}_{rr^{\prime }k}\right)\leq z_{r^{\prime }k}^{u} \end{array}$$

(8) $$\begin{array}{l} \forall p\in P_{rr^{\prime }},\forall r\in R_{s},\forall r^{\prime }\in R_{e},\forall k\in K \end{array}$$

Constraint (1) ensure that each transport request is assigned only one time and can be followed by another request. Constraint (2) makes sure that each charging request is presented by at most one AGV. Each charging request *r* has only one path. Constraint (3) ensures that the capabilities of the requests and AGVs match. Constraint (4) ensures that self-visits are avoided. Constraints (5) and (6) make sure that the number of entering paths of request, execution paths, and leaving paths of each request are consistent. The time constraints are given by (7) and (8), and Constraint (7) calculates the travel time from the pickup node of request *r* to the delivery node. Constraint (8) calculates the travel time between different requests. The travel time includes transportation time and delay time, where *M* is a large positive constant.


(9) $$\begin{array}{l} b_{l}^{k}\leq \lambda_{rk}^{d}\leq b_{h}^{k}\forall r\in R_{s},\forall k\in K \end{array}$$

(10) $$\begin{array}{l} b_{l}^{k}\leq \lambda_{rk}^{u}\leq b_{h}^{k}\forall r\in R_{e},\forall k\in K \end{array}$$

$$\begin{array}{l} \lambda_{rk}^{u}+d_{k}\left(z_{rk}^{d}-z_{rk}^{u}\right)-M\left(1-{{\text{x}}^{p}}_{rk}\right)\leq \lambda_{rk}^{d} \end{array}$$

(11) $$\begin{array}{l} \forall p\in P_{r},\forall r\in T,\forall k\in K \end{array}$$

$$\begin{array}{l} \lambda_{rk}^{d}+d_{k}\left(z_{r^{\prime }k}^{u}-z_{rk}^{d}\right)-M\left(1-{{\text{y}}^{p}}_{rr^{\prime }k}\right)\leq \lambda_{r^{\prime }k}^{u}\forall p\in P_{rr^{\prime }}, \end{array}$$

(12) $$\begin{array}{l} \forall r\in R_{s},\forall r^{\prime }\in R_{e},\forall k\in K \end{array}$$

The constraints related to power consumption are given by (9) to (12). Constraints (9) and (10) set the lower and upper bounds for an amount of battery discharge. Constraint (11) calculates the amount of battery discharge due to the travels between the source and destination of a request. Constraint (12) calculates the amount of battery discharge due to the travel between the destination and the source of two requests.


(13) $$\begin{array}{l} {x^{p}}_{rk}\in \left\{0,1\right\}\forall p\in P_{r},\forall r\in R,\forall k\in K \end{array}$$

(14) $$\begin{array}{l} {y^{p}}_{rr^{\prime }k}\in \left\{0,1\right\}\forall p\in P_{rr^{\prime }}\forall r\in R_{s},\forall r^{\prime }\in R_{e},\forall k\in K \end{array}$$

(15) $$\begin{array}{l} z_{rk}^{u}\geq 0\forall t\in R_{e},\forall k\in K \end{array}$$

(16) $$\begin{array}{l} z_{rk}^{d}\geq 0\forall t\in R_{s},\forall k\in K \end{array}$$

The valid domains of the binary variables are given by constraints (13)–(16), which guarantee valid domains for the other decision variables.

## 4. Hierarchical planning method

In this section, a hierarchical planning method is proposed to solve the joint task assignments, path planning, and conflict problem for just-in-time scheduling. This method is inspired by the work of Hooker and Ottosson (2003) and decomposes the integrated optimization problems into an aggregated upper-level master problem and a lower-level subproblem. The upper-level problem is to make decisions for AGV task assignment/sequence, which determines a candidate elite solution set where the collision constraints for AGVs are neglected. The lower-level subproblem is to solve the optimal path planning problem with collision constraints under the conditions of the tentative solution at the upper level. The conflict problem is considered in both the master problem and subproblem, the collision between AGVs can be reduced by changing the detailed paths for vehicles or the scheme of task assignment and sequence. In summary, the objective is to minimize the AGV transportation time, which is the sum of the total travel time and the delay time (waiting or detour time for avoiding collisions). The detailed steps are described as follows:

Step 1. The upper level: Task assignment and sequence to AGVs where the collision constraints are removed from the original problem, and the transportation time of each task for each AGV is defined as the minimal time from the starting node to the delivery node. The master problem is regarded as the task assignment/sequence problem with constraints such as heterogeneous AGVs and batteries. In this study, a tentative elite solution set φ_*i*_ sorted in the ascending order of the objective function value is generated by HDSTA where the solution in the elite solution set is denoted by *p*_*n*_.

Step 2. The lower level: Select the specific paths to perform the assigned tasks for the AGVs under the condition that a tentative solution *p*_*n*_ is derived from a master problem. For each solution, *p*_*n*_ ∈ φ_*i*_ selected in the ascending order, the subproblem, which is concerned with the path planning problem to select the optimal paths with collision constraints for AGVs, is solved by the select procedure, while a list of conflict results with memories is generated, called Tabu List Λ_*i*_. If the result of the selected procedure is conflict-free paths (termination criterion 1), the algorithm is completed; otherwise, recording conflict results to Λ_*i*_. In the iteration, the solution with the minimum objective function values is recorded as the tentative optimal solution *p*_*best*_ and its delay time is defined by *t*_*p*_.

Step 3. Algorithm termination criterion 2: The maximum allowable delay time is defined by ε. If *t*_*p*_ derived in Step 2 is less than ε, the algorithm is completed.

Step 4. Regenerate the tentative elite solution set φ_*i*_ considering the information is recorded in the tabu list Λ_*i*_ by HDSTA. If there is no improvement in the objective function value after five iterations, the algorithm is completed (termination criterion 3); otherwise, updating Λ_*i*_ and returning to Step 2.

The main scheme of hierarchical planning is illustrated in Algorithm 1. The accurate information about all AGVs and tasks are known, and the path expert database must be computed offline in advance.

**Algorithm 1:** Main scheme of hierarchical planning.

**Require:** set of AGVs *K*, set of tasks *T*, path expert database *N*_*s*_
1: **if** there is a change in transport or charging requires **begin**
2: **Extract** finished tasks from the running schedule
3: **Combine** the remaining tasks and new tasks
4: **Update** *K*, *T*
5: **end**
6: **Initialize** tentative elite solution set φ_*i*_ by HDSTA
7: **while** (*y*_1_ = 1 **or** *y*_2_ = 1**or** *y*_3_ = 1)
8: **for each** *p*_*n*_ ∈ φ_*i*_
9: [*p, T, t*_*p*_, *S*_*p*_] ← SP (*p*_*n*_)
10: **if** *t*_*p*_ = 0 then
11: *D* ← *p*_*n*_; *P*_*best*_ ← *p*; *T*_*best*_ ← *T*; *y*_1_ ← 1
12: **break for**;
13: **end if**
14: **if** *t*_*p*_ < *t*_*best*_ then
15: t_*best*_ ← *t*_*p*_;*P*_*best*_ ← *p*; *T*_*list*_ ← [*p*_*n*_, *S*_*p*_];*D* ← *p*_*n*_;*T*_*best*_ ← *T*
16: **end if**
17: **end for**
18: **if** *t*_*best*_ < ε then
19: *y*_2_ ← 1
20: **end if**
21: **if** *y*_1_ = 0 **or** *y*_2_ = 0 then
22: **update** the elite solution set φ_*i*_ by HDSTA
23: **update** *l*
24: **if** *l* > 5
25: *y*_3_ ← 1
26: **end if**
27: **end if**
28: **end while**
29:*S* ← (*D, P*_*best*_)
**return** *S*

An HDSTA with a path-select procedure and tabu list is proposed to find the optimal solution. The algorithm starts with a dynamic serve framework by generating a reschedule at the appropriate time interval methodology, based on the concept of dynamic scheduling, when there is a requirement for an additional task or AGV charging (lines 1–5). Then, the initialization of the tentative elite solution set φ_*i*_ using HDSTA (line 6) was carried out. For each solution *p*_*n*_ ∈ φ_*i*_, detailed paths are generated using the path select procedure (SP). The transport time, delay time, and conflict points are calculated, and a tabu list is generated (lines 8–17). If a solution exists in the elite solution set that conflict-free paths can be generated in path planning is marked as *y*_1_(lines 10–13). The optimal solution in the elite solution set whose delay time is less than ε is marked as *y*_2_ (lines 18–20). If the objective function values showed no improvement after multiple iterations are marked as *y*_3_ (lines 24–26), the iteration of the elite solution set is updated by HDSTA by incorporating the Tabu List constraints until one of the termination conditions are met and generating an integrated scheduling solution containing the sequential assignment solution and the detail path solution.

### 4.1. HDSTA

**Figure 2:**
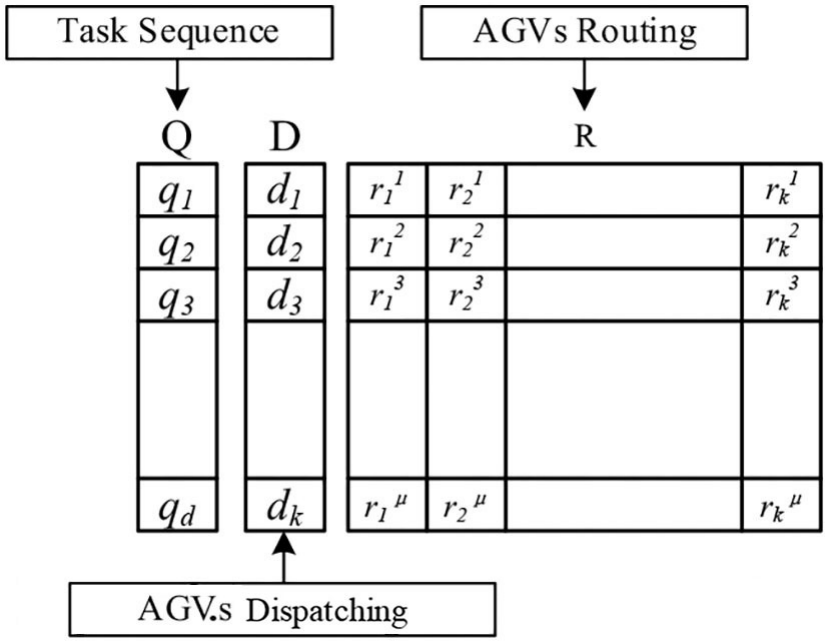
State space representation.

The state transition algorithm (STA) (Yang et al., 2013) is a kind of intelligent optimization algorithm originally proposed by Zhou et al. (2012) with good global search capability and convergence property. In our proposed HDSTA, the integrated problem is decomposed into individual elements and the individual S is defined by three “state spaces” related to its tasks sequence, AGV dispatch, and the corresponding routes, as depicted in Figure 2.

The task sequence state space *Q* registers for each task. The space *Q* consists of some types of tasks, with the subspace *q*_*d*_ ∈ *Q* representing one type of task collection. The first type is charging requirement, while the others depend on the number of heterogeneous AGV types. It is worth mentioning that, to ensure performing urgent tasks first, the sequence of the task in each subspace must observe the rule of task priority. The dispatching state space *D* contains the task assignment for all AGVs, and the subspace *d*_*k*_ ∈ *D* represents the dispatching of AGV *k* ∈ *K*. Finally, the AGV routing *R* corresponds to all paths, while the subspace $r_{k}^{u_{k}}\in R$ represents the path of the *u*th task of the *k*th AGV, respectively.

The integrated scheduling state space of AGVs is decoded for three subproblems, namely, task sequence, dispatching, and routing. Regularly representing an individual solution with appropriate random numbers is a very effective method to solve combinatorial optimization problems. However, to solve scheduling optimally in an integrated manner, intrinsic connections and constraints between the subproblems must be established.

**Figure 3:**
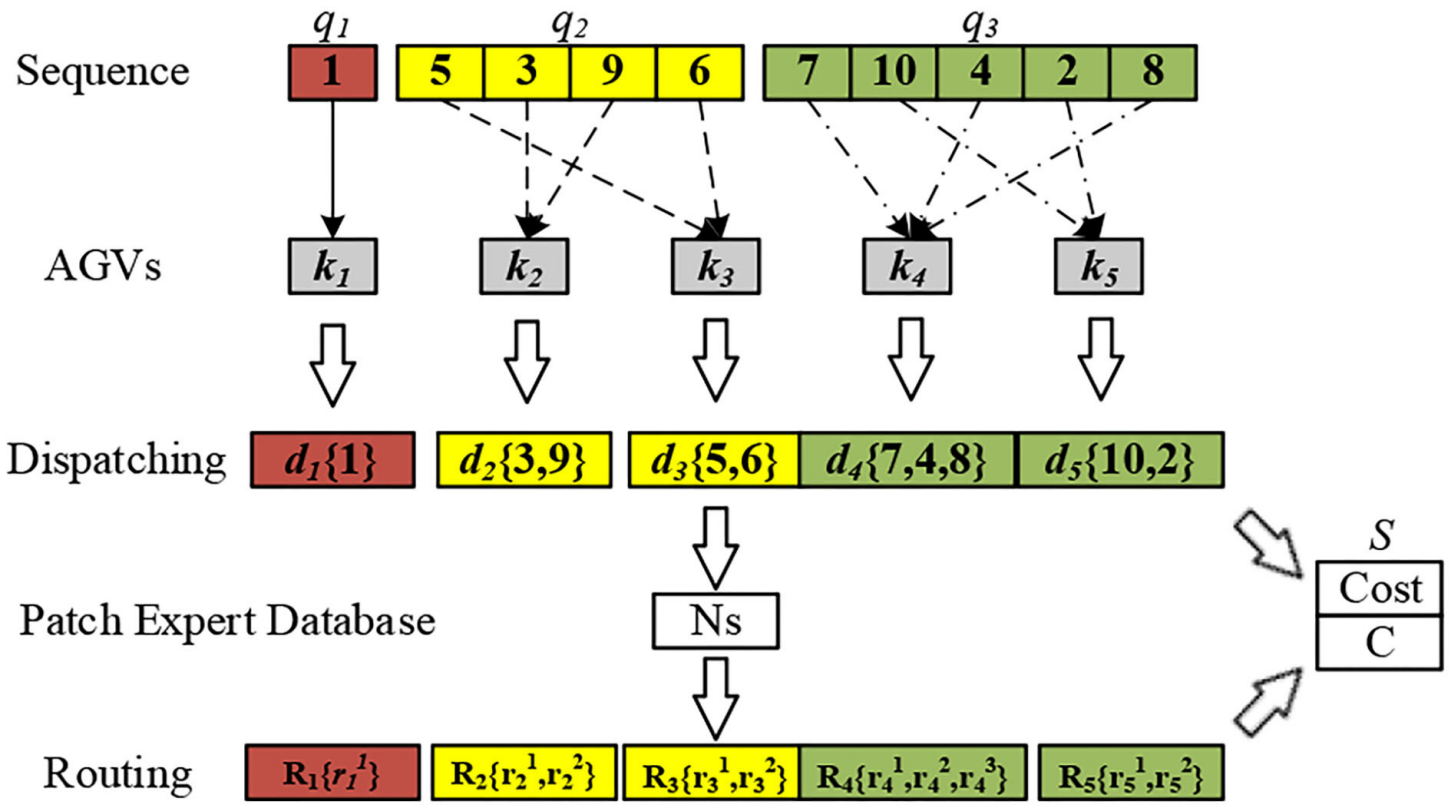
A description of the integrated scheduling problem.

An illustration of the integrated method is given in Figure 3. A sequence space consists of three types of requirements: a charging request (denoted in red), a piggyback transportation requirement (denoted in yellow), and a pallet transportation requirement (denoted in green). By randomly assigning tasks in each subspace to the matching AGVs, the corresponding dispatching space is generated. Each time the generated dispatching space performs the routing procedure, that is, to select the optimal path with the least collision in the path expert database and generate the conflict result feedback *C*. The optimization objective result *S* is the sum of total travel time *Cost* based on the current solution of dispatching space and conflict result *C*. The feasible solution of scheduling consists of a dispatching scheme and a detailed routing scheme. The role of sequence space is to define various requirements with priority and increase the search range of the algorithm.

In the task sequence state space *Q*, a candidate solution set is generated by the three special operators, a swap operator, shift operator, and symmetry operator (Yang et al., 2013), which are very effective to solve discrete optimization problems. Moreover, a candidate solution set is created by the times of the transformation called the search enforcement (SE) and the translation operator is performed only if a better new trail is found.

**Figure 4:**
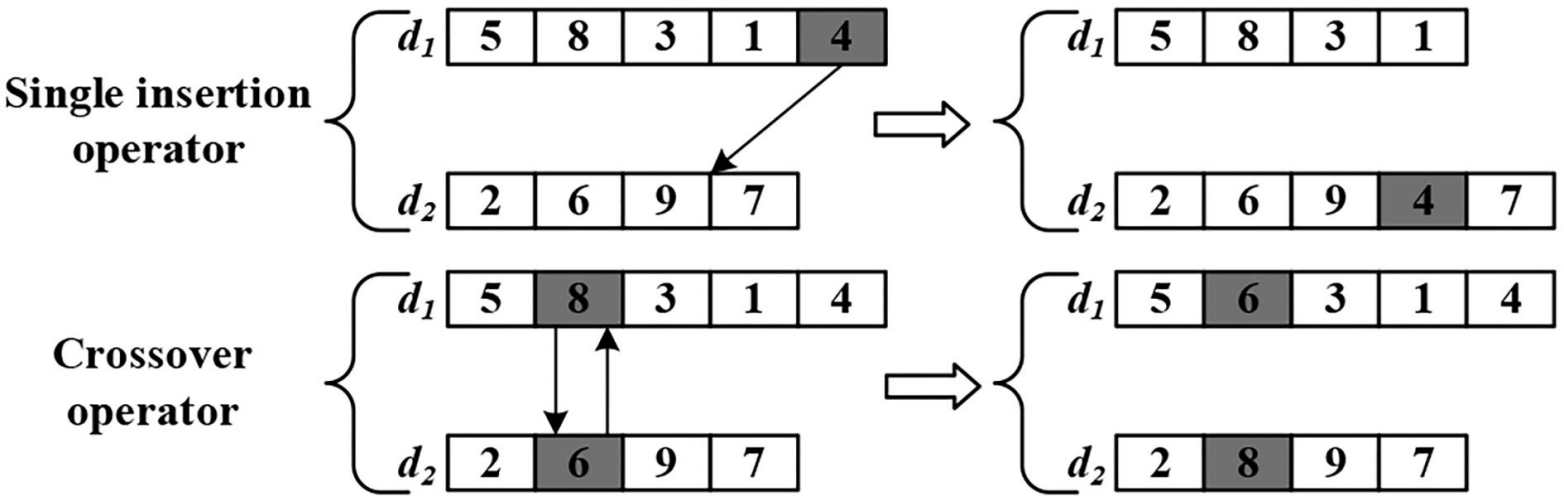
A description of move operators.

In the AGV dispatching space, the same four operators are applied to produce a candidate solution set, which is referred to as self-learning. However, the search space of the basic state transformation algorithm is normalized or specialized and cannot directly solve the problem with multiple subspaces. In the dispatching space, the communication strategy between the subspace *d*_*k*_ is necessary to exchange information for increasing the search intensity. Thus, we employed two move operators, illustrated by Figure 4. (1)—the single insertion operator (SI): displaces the last task element of one subspace of dispatching to a random position in another subspace of dispatching and (2) the position-based crossover operator (PBC): exchange task elements of the same position randomly in two different subspaces of dispatching.

### 4.2. Select procedure

The method of AGV routing based on the path expert database is proposed for the first time. In the industrial context, collisions can be avoided by hardware, and conflict-free path planning is not our purpose as it takes a lot of computing time and easily falls into locking in large-scale problems. The proposed method is to select a detailed path with the least conflicts from the path expert database according to the current dispatching and recording of the information of the conflict.

In this section, we give a path expert database *N*_*s*_ and task assignment for all AGVs, *D* = *d*_1_, *d*_2_, *d*_3_ … *d*_*k*_ in this section. Let *T*_*c*_ denote the punishment time of the conflict process. As mentioned earlier, each task *t* consists of a pickup request $o_{u}^{t}$ and a delivery request $o_{d}^{t}$. To represent a path-planning solution, we assign to the route *r*_*t*_ of each task two paths from the path expert database. The $r_{u}^{k}$ and $r_{u^{\prime }}^{k}$ are said to be traveling paths for pickup and delivery, respectively, by AGV *k*, and the *n*th path of the route *r*_*ij*_ from depot *i* to depot *j* in the path expert database is defined as *Path*(*i, j, n*) ∈ *N*_*s*_. The selection procedure for AGV routing to optimize path planning and obtain punishment time is shown in Algorithm 2.

**Algorithm 2:** Select procedure for AGV routing.

**Require:** path expert database *N*_*s*_, dispatching *D*
1: *T*_*c*_ ← 0
2: **repeat**
3: **for** each *d*_*k*_ ∈ *D*
4: **for** each *t* ∈ *T*_*k*_
5: $r_{u}^{k}$ ← *Path*($o_{u}^{t}$, 1); $r_{u^{\prime }}^{k}$ ← *Path*($o_{d}^{t}$, 1)
6: *r*_*t*_ ← $r_{u}^{k}$; *r*_*t*_ ← $r_{u^{\prime }}^{k}$; *R*_*k*_ ← *r*_*t*_
7: **end for**
8: *R* ← *R*_*k*_
9: **end for**
10: [*T*_*c*_, *C*_*i*_] ← Con (*R*)
11: **if** *T*_*c*_ ≠ 0
12: **for** each *C*_*i*_
13: *R*′ ← Replace (*R*_*k*_)
14: $T_{c}^{{\;}^{\prime }}$ ← Con (*R*′)
15: **if** $T_{c}^{{\;}^{\prime }}$<*T*_*c*_
16: *T*_*c*_ ← $T_{c}^{{\;}^{\prime }}$
17: *R* ← *R*′
18: **end if**
19: **end for**
20: **end if**
21: **return** *T*_*c*_, *R*

Set *T*_*c*_ to zero at the beginning (line 1). Then, a loop is executed to assign paths to each subspace *d*_*k*_ (lines 3–9). Each task *t* ∈ *T*_*k*_ is assigned to the first path in the path expert database by the loop (lines 4–7). The time coordinates and punishment time of conflicts are calculated based on the time window, referred to as “CON” (line 10). When the conflict time is not zero, replace the best path with another path in the path expert database, referred to as “Replace,” and update the routing data if the new path is better than all the queried paths (lines 11–20). Finally, the procedure returns the best solution (line 21).

In the dispatching space, the computational procedure does not consider the conflict situation. Thus, we establish a tabu list to record the conflict situation and then feed it back to dispatching and eliminate the unfeasible solutions in the next state to reduce conflicts whenever possible.

For example, in the current dispatching space, subspace *d*_1_{2, 1, 3, 5} and subspace *d*_2_{8, 7, 6, 4} have a conflict in the routing procedure and the conflict situation is for AGV 1 in performing task 2 and AGV 2 in performing task 8. The conflict results *d*_1_{2, *x*, *x*, *x*} and *d*_2_{8, *x*, *x*, *x*} are recorded for infeasible solution domains, where *x* is an arbitrary task. The result represents the infeasible solution that AGV 1 first performs task 2 while AGV 2 first performs task 8. In the following stage, the infeasible solutions are removed.

### 4.3. Path expert database

To the best of our knowledge, the establishment of the path expert database in the offline state for path planning is proposed for the first time. The optimal path between depots is several; besides, there are many good paths as well.

The concept of a path expert database is a collection that contains all optimal paths and good paths with sequences between depots for path replacement in case of a conflict.

The path expert database can be established by manually experience or algorithm programs depending on the different specifications of the warehouse. In this section, we propose an improved A^*^ algorithm to generate a path expert database as shown in Algorithm 3.

**Algorithm 3:** Improved A^*^ algorithm for establishing the path expert database.

**Require:** graph *G*, set of depots *X* = {*x*_1_, *x*_2_, *x*_3_ … *x*_*s*_}
1: **Initialize** matrix *H*_*s*_
2: **repeat**
3: CLOSE list ← 0; final ← 0; Mark = ∅
4: OPEN list ← start
5: **while**
6: **while** OPEN list ≠ 0
7: **if** the number of the latest node in OPEN list = 1
8: Current node ← the latest node in OPEN list
9: **else** Current node ← the first node
10: Mark ← OPEN list; Mark ← CLOSE list
11: **end if**
12: **if** current node = end point
13: break final ← CLOSE list
14: **end if**
15: **for each** neighbor (Current node)
16: **if** neighbor ∉ Obstacle
17: new cost = *f*(neighbor)
18: **if** new cost < cost allel neighbor ∉ CLOSE list
19: OPEN list ← neighbor
20: **else** CLOSE list ← neighbor
21: **end if**
22: CLOSE list ← current node
23: **end if**
24: **end for**
25: **end**
26: **if** Mark ≠ ∅
27: OPEN list ← Mark (*i*); CLOSE list ← Mark (*j*)
28: **else** break
29: **end if**
30: **end**
31: final = sort (final)
32: *N*_*S*_ ← final
33: **until** the specified termination criterion is met
34: **return** *N*_*S*_

The initial matrix of depots is defined by *H*_*s*_ (line 1). The algorithm loops over each route *r* ∈ *H*_*s*_. A loop program calculates the paths between each depot (lines 2–33). Let the CLOSE list and the OPEN list denote a collection of nodes that have already been estimated and the collection of nodes that waiting for estimating. The path result is recorded in “final” and the points of the same valuation are recorded in “mark” (lines 3–4). The algorithm executes a loop that finds the optimal path between the two depots based on the A^*^ algorithm and records the other points of the same valuation in each iteration (lines 6–25). If the collection “mark” is not blank, remount the information of points recorded successively to find all good paths between two depots (lines 26–29)—a record of all the path results (lines 31–32) and the procedure returns path expert database *N*_*S*_ finally (line 34).

## 5. Computational experiments

**Figure 5:**
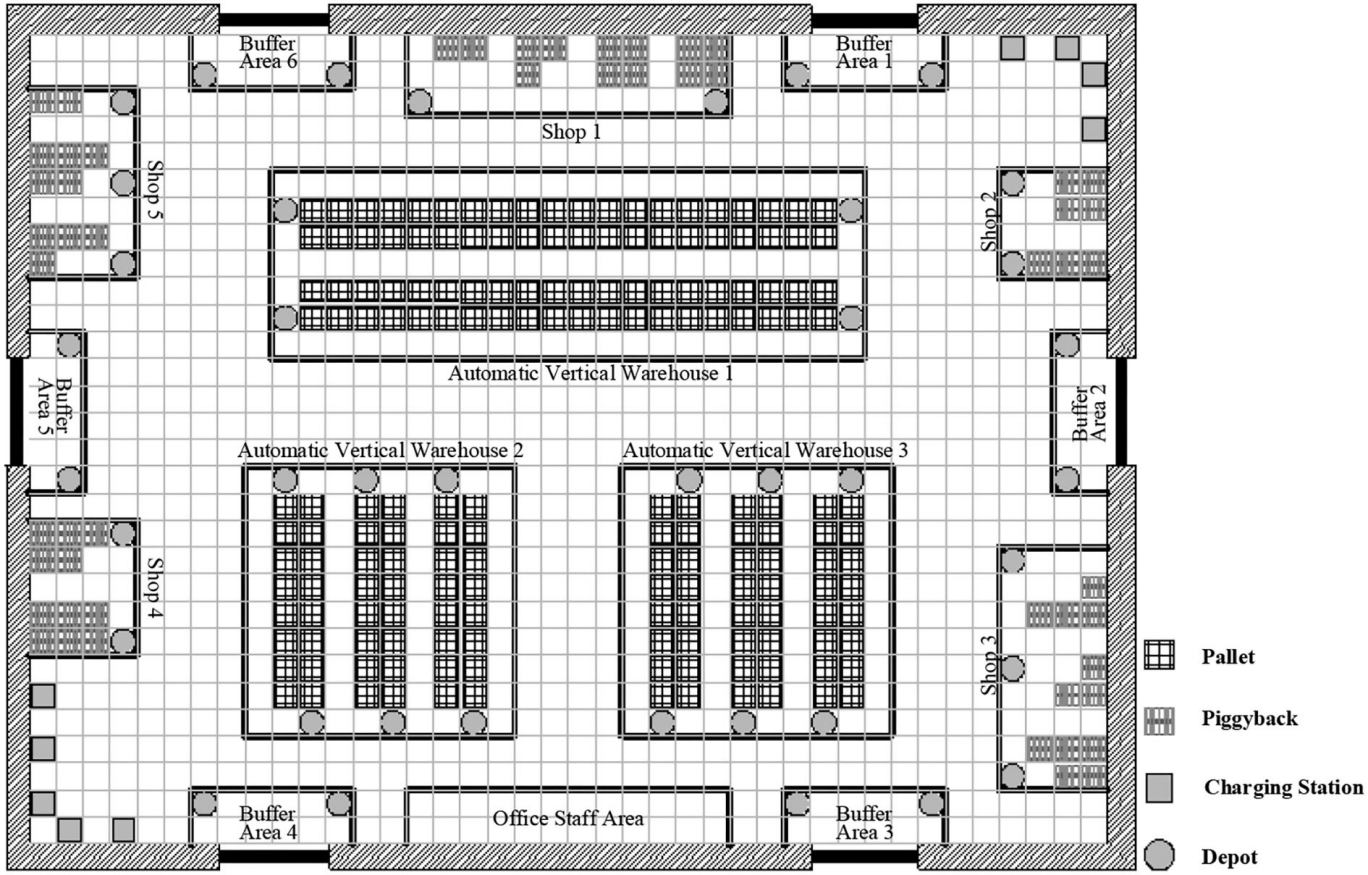
Warehouse layout.

To evaluate the performance of the proposed method, computational experiments are performed in a dynamic scenario and under different scenarios with varying fleet sizes and numbers of tasks. We implemented the proposed dynamic scheduling method on a computer with an Intel (R) Core (Tm) CPU i7-9700 4.8 GHz and 8 GB RAM with a 64-bit Windows 10 operation system, while the scheduling rule is implemented in Python v3.6. The study adopts the warehouse production data located in Changsha, China. The layout of this warehouse is illustrated in Figure 5, which consists of 12 buffer area depots, 12 shop depots, 15 automatic vertical warehouse depots, and 5 charging stations. From the feedback from the practitioners, the average number of requests waiting for assigning is about 30 in a horizon, a horizon with more than 60 requests is regarded as a busy period.

The position of the depots (or stations) in the layout is fixed. Therefore, we made use of a distance matrix to compute the travel time of AGVs. We generated a path expert database through the program while offline and also note again that the collision-free trajectories are not considered in our experiments, since those collisions between the AGVs can be avoided by hardware.

### 5.1. Dynamic scheduling

**Figure 6:**
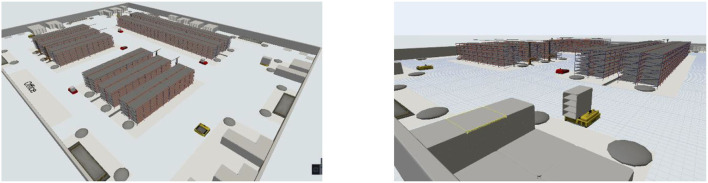
FlexSim-based digital simulation system.

A FlexSim-based digital simulation system is established to dynamically analyze the operation of AGV systems under the industrial warehouse instances, as shown in Figure 6.

The description of the dynamic scheduling problem is shown in Table 1. The integrated scheduler algorithm processes a total of 60 tasks arriving at three different random intervals of time. Initially, 25 tasks are scheduled. While executing the initial schedule, 15 new tasks are added to the system at time *t* = 14 min. This results in a dynamic rescheduling of the system. While executing the current schedule, 20 more new tasks were added to the system at time *t* = 22 min.

**Table 1:** The description of the dynamic scheduling problem.

**Schedule**	**Start time (min)**	**New tasks**
Initial	0	Task 1, Task 2, …, Task 25
Interrupt 1	14	Task 26, Task 27, …, Task 40
Interrupt 2	22	Task 41, Task 42, …, Task 60

**Figure 7:**
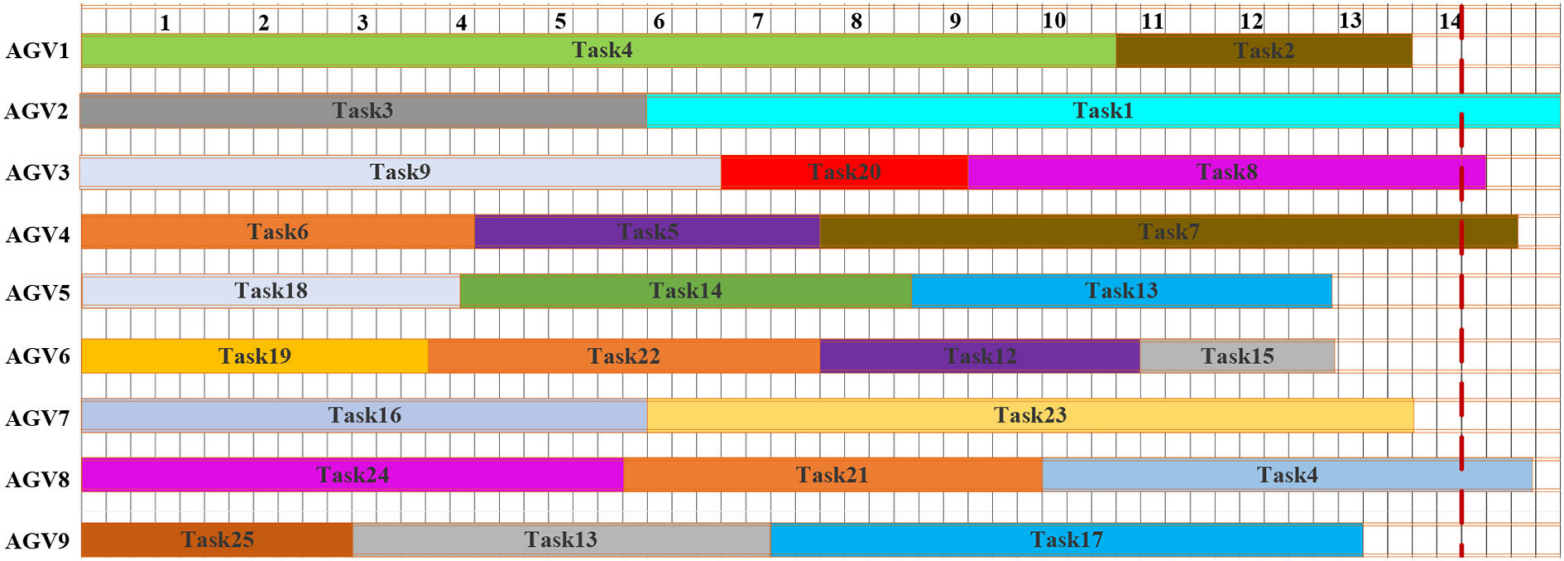
Gantt chart for dynamic scheduling 1.

**Figure 8:**
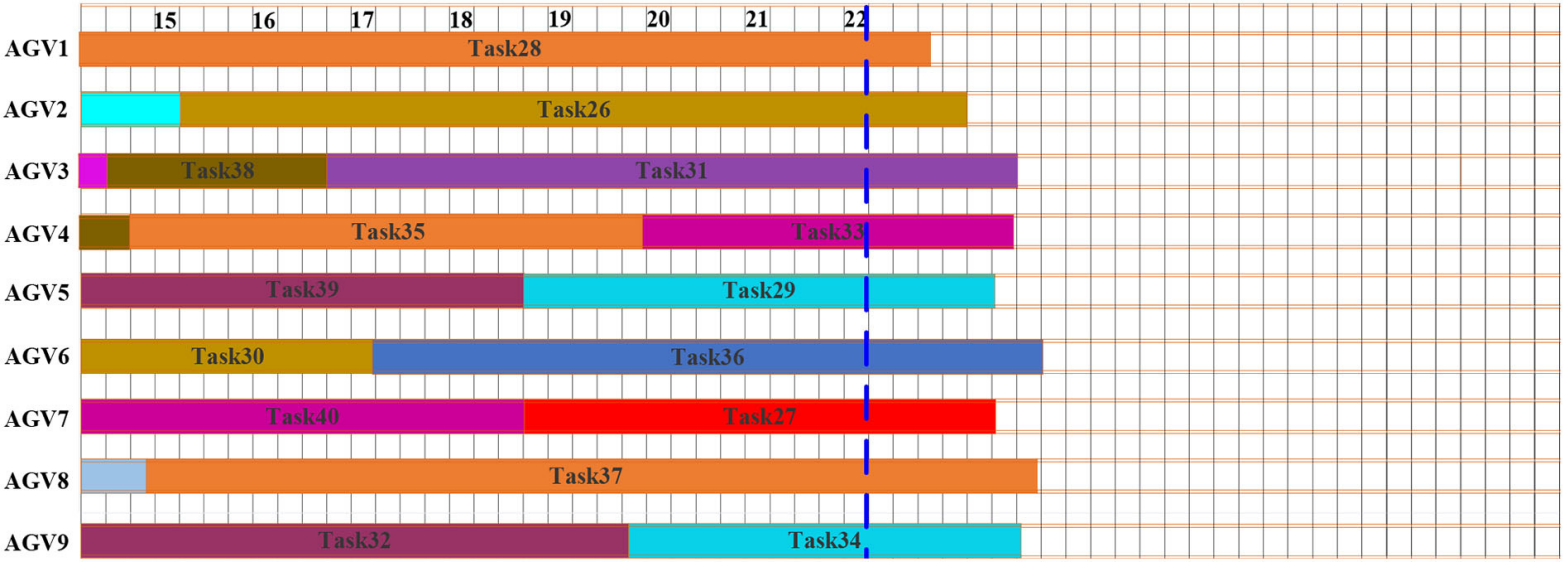
Gantt chart for dynamic scheduling 2.

**Figure 9:**
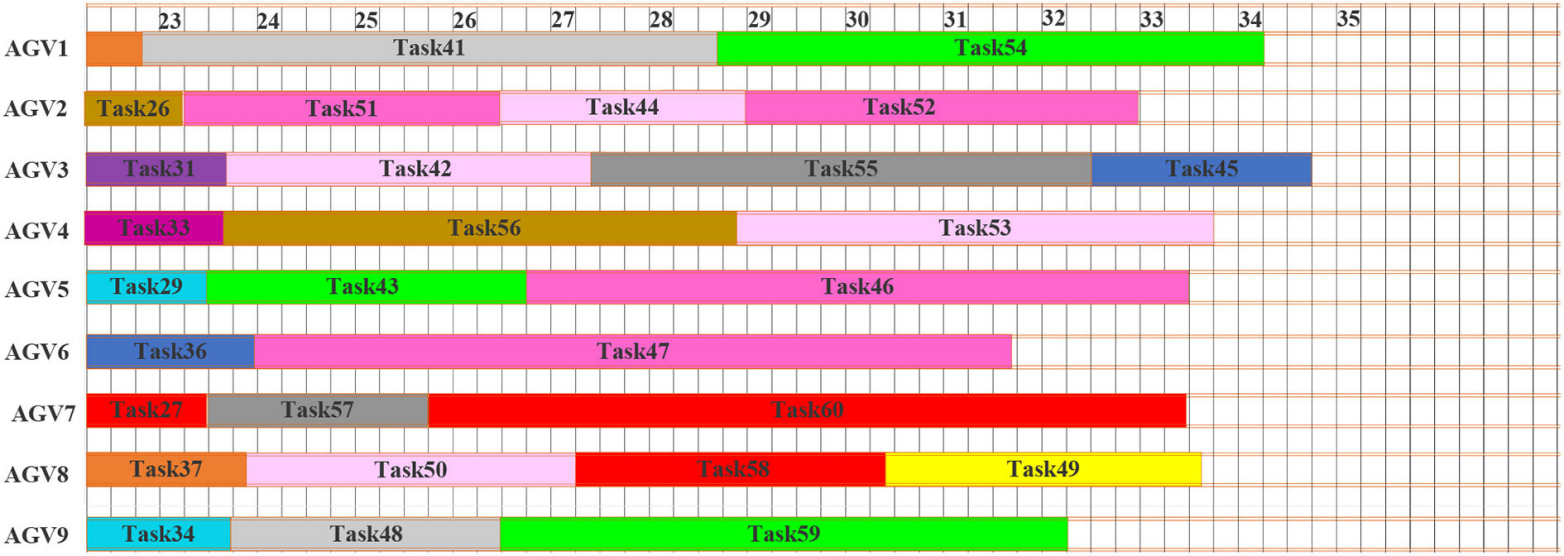
Gantt chart for dynamic scheduling 3.

As stated in the methodology, this is based on the concept of scheduling and rescheduling under an appropriate time intervals methodology of dynamic scheduling. Figure 7 shows the Gantt chart of the initial schedule. The dashed line at time *t* = 14 min represents the interruption and rescheduling, the points when new tasks are added to the system. The uncompleted tasks currently at the execution stage at the interruption and the rescheduling points are task 1, task 8, task 7, and task 4. The tasks in execution will continue with the preemption in the next planning time horizon until the operation is completed. Figure 8 shows the Gantt chart for the generated new schedule, in which the new tasks are added after the interruption of the previous schedule. The operations at tasks 1, 8, 7, and 4 marked by the parallel slanted lines are the remaining operation from the previous schedule. On this schedule, all tasks in the system are either completed or the last task is under execution before the interrupt point in time *t* = 22 min. Figure 9 shows the Gantt chart for the generated new schedule. The tasks completed at the current interrupt and the rescheduling point are tasks 28, 26, 31, 33, 29, 36, 27, 37, and 34. Dynamic path planning adjusts the path without interrupting the current task execution process.

### 5.2. Analysis of the scheduling results

**Figure 10:**
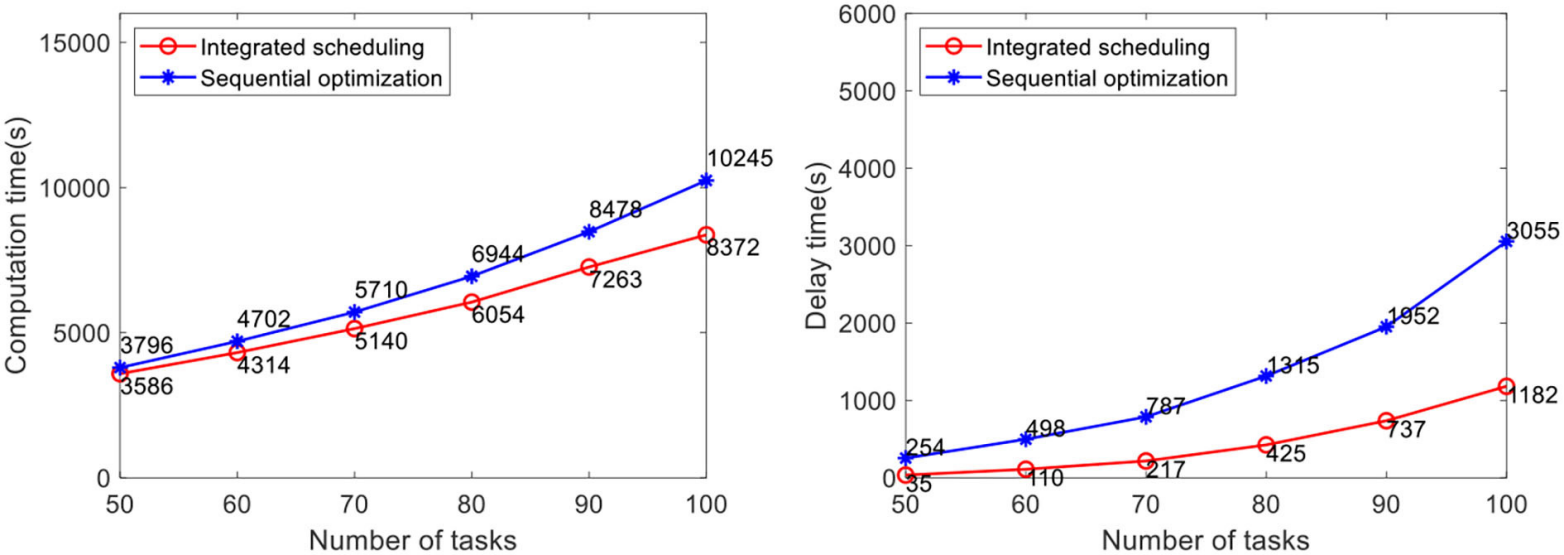
Comparative analysis of task completion time and delay time with 5 AGVs.

**Figure 11:**
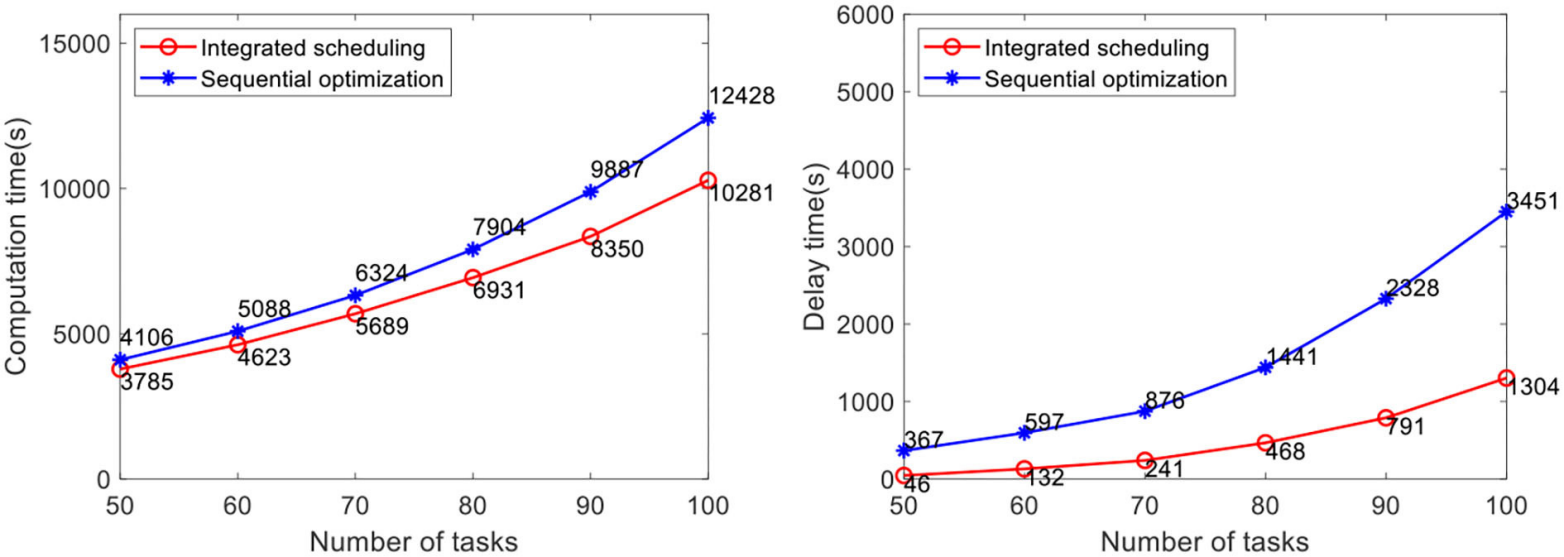
Comparative analysis of task completion time and delay time with 10 AGVs.

**Figure 12:**
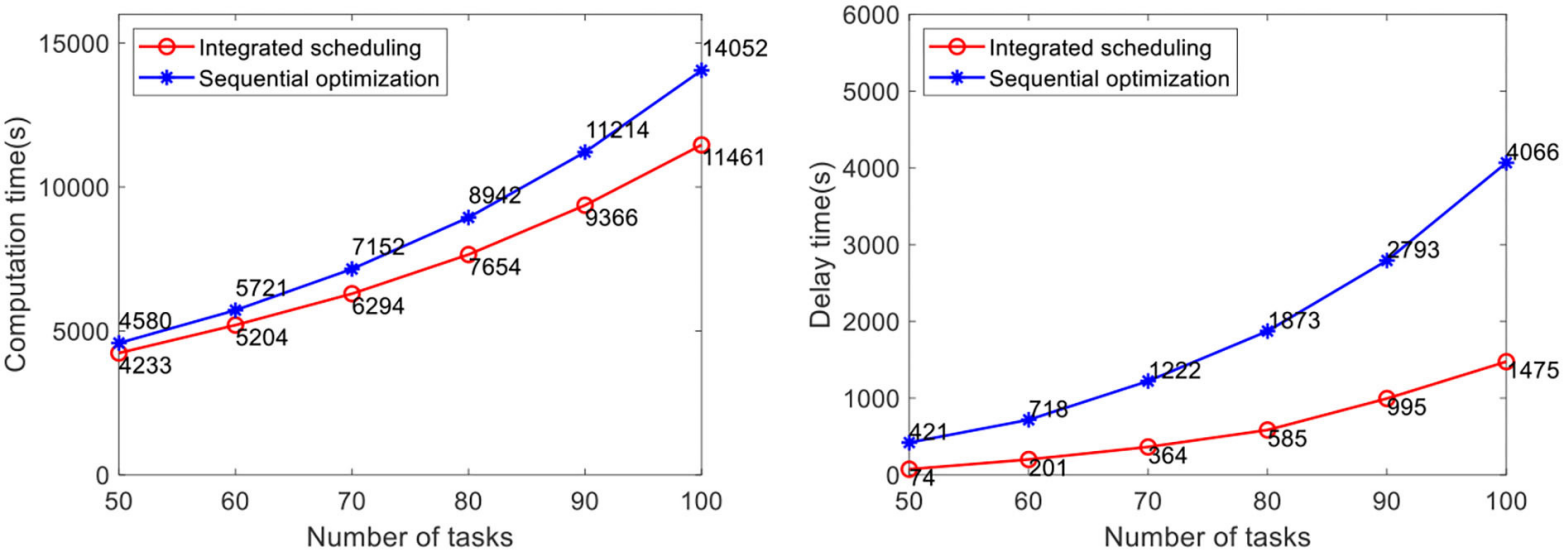
Comparative analysis of task completion time and delay time with 15 AGVs.

The efficacy of our method is verified by computational experiments using real-world data with varying fleet sizes and numbers of tasks. The number of AGVs to be dispatched is 5, 10, and 15, respectively. The number of tasks to be allocated in the case is 50, 60, 70, 80, 90, and 100, respectively. Each case randomly generates five groups of tasks and runs them 10 times, for a total of 50 runs of the program. The average value is taken as the result. At present, the advanced AGV systems in industrial warehouses adopt the scheduling method of sequential optimization, of which the method proposed by Lian et al. (2020) is the most representative. Therefore, this method is selected for comparative verification of the analyses of real warehouse cases. Problems not considered in this method, such as the heterogeneity of the AGVs and battery constraint, are improved before the comparative verification in this study. In the case study, the comparison results of the task completion time and the delay time of the two methods are shown in Figures 10–12.

The results show that the integrated scheduling method proposed in this study has better performance and better solutions are found in all cases. In particular, the average task completion time is 13.62% less and the average delay time is 76.69% less than the sequential optimization of the scheduling method. The average delay time difference between the two methods is only 219 s when the number of tasks is 50 using 5 AGVs, but it increases to 3,591 s when the number of tasks increases to 100 using 15 AGVs. With the increase in task scale, the probability of conflicts between AGVs also increases dramatically. The sequential optimization scheduling method cannot avoid the impact of conflicts from the task allocation process, while the proposed integrated scheduling can avoid most conflicts by changing the task assignment and specific execution path.

### 5.3. Comparison of algorithms

To verify the performance of HDSTA, a larger-scale case template needs to be established. We generate 10 instances for each scenario with 50–150 tasks and AGVs to be scheduled, ranging from 5 to 25; each instance runs ten times to compute the mean. In each instance, HDSTA with the adaptive large neighborhood search algorithm (HALNS) (Dang et al., 2021) was compared with the preplanning algorithm (PPA) (Maza and Castagna, 2005). HALNS is a hybrid algorithm of the adaptive large neighborhood search algorithm and the linear programming algorithm, which is proposed to solve the heterogeneous AGV scheduling problem with charge capacity constraints. However, this method does not consider the problem of conflict and deadlock. For comparison, we developed the conflict detection method to compute the delay time of its optimal solution. PPA is a strategy to generate conflict-free paths.

Table 2 compares the task completion time and scheduling computation time for varying scenarios with different numbers of AGVs and different numbers of tasks, where the task completion time is directly proportional to the operation cost, which can visually reflect the collaborative operation efficiency of AGVs, and the computation time is an important index of dynamic scheduling, which can reflect the computation efficiency of AGV systems. Table 2 compares the performance and computation time of PPA, HALNS, and HDSTA for 150 sets of tasks in 15 case types. For example, when the task volume is 50 and the number of AGVs is 5, 9, and 11, respectively, the task completion times of the scenarios calculated by optimal scheduling with the HDSTA algorithm are 3,738, 3,811, and 3,845 s and the computation times are 2.23, 2.33, and 2.37 s, respectively. The results of 150 sets of tasks for 15 case types are analyzed and compared with PPA and HALNS. The average task completion time of the HDSTA solution proposed in this study is lower by 3.44 and 7.27% and the computation time is less by 84.15 and 81.92%.

**Table 2:** Results of the varying scenarios.

**NO**.	**Number of tasks**	**Number of AGVs**	**PPA**		**HALNS**		**HDSTA**	
			**Computation time (s)**	**Task completion time (s)**	**Computation time (s)**	**Task completion time (s)**	**Computation time (s)**	**Task completion time (s)**
1	50	5	7.61	3,747	4.24	3,744	2.23	37,38
2	50	9	7.73	3,830	4.36	3,862	2.33	3,811
3	50	11	8.13	3,853	4.68	3,876	2.37	3,845
4	60	3	11.41	4,522	6.88	4,529	2.84	4,522
5	60	8	12.14	4,615	7.20	4,628	2.83	4,608
6	60	13	12.81	4,693	8.36	4,854	2.90	4,685
7	80	4	19.91	6,570	16.07	6,569	3.55	6,558
8	80	6	22.89	6,683	18.15	6,711	3.91	6,654
9	100	7	38.92	8,421	31.94	8,408	5.64	8,311
10	100	15	43.85	8,636	30.11	8,756	6.22	8,541
11	120	15	50.21	12,850	41.65	13,365	8.60	12,305
12	120	18	54.33	12,902	56.84	14,025	8.84	12,654
13	120	19	55.12	13,357	58.52	14,359	8.92	13,147
14	150	22	98.21	18,724	84.74	19,015	13.45	17,521
15	150	23	116.89	19,031	91.61	19,584	14.16	16,984

## 6. Conclusion

This article studied the problem of scheduling a heterogeneous fleet of AGVs. A MILP model was formulated to minimize the sum of the costs of requests and the tardiness costs of conflicts. The hierarchical planning method is used to decompose the complex and integrated scheduling problem. We propose that HDSTA combine select procedures. The major novelty of this study is the ability to solve the dynamic integrated scheduling problem for heterogeneous AGV fleets with battery constraints. We performed numerical experiments to validate our model according to the real-world conditions of the automated warehouses in Changsha, China.

In the future, we may extend our research to improve our approach to multiple pickups and deliveries along the same route (multi-load AGVs) and the inclusion of path planning in the scheduling process.

## Data availability statement

The original contributions presented in the study are included in the article/supplementary material, further inquiries can be directed to the corresponding authors.

## Author contributions

EH and SS: conceptualization and writing—original draft preparation. EH: data curation. EH and JH: methodology, validation, and formal analysis. JH: writing—review and editing and funding acquisition. All authors contributed to the article and approved the submitted version.
